# Resolution of Corneal Dellen After an Uneventful Pterygium Surgery with Punctal Cautery

**DOI:** 10.7759/cureus.8250

**Published:** 2020-05-23

**Authors:** Bharti Sharma, Sushil Kumar Bajoria, Abhishek Patnaik, Ravi Barbhaya

**Affiliations:** 1 Ophthalmology, Tata Main Hospital, Jamshedpur, IND; 2 Ophthalmology, RDB Eye Care, Jamshedpur, IND

**Keywords:** dellen, pterygium, fibrin glue, punctal cautery, tear film, conjunctival limbal autograft

## Abstract

Corneal dellen are a rare and serious complication after primary pterygium surgery with conjunctival limbal autograft (CLAG) with fibrin glue without antimetabolites. Dellen are caused by interruptions of the tear film and local dehydration of the cornea. If untreated, they may lead to corneal perforation. We describe the case of a patient who developed corneal dellen 15 days after uneventful pterygium excision with CLAG with fibrin glue without the use of antimetabolites. There was no satisfactory response to lubricants and patching, and the patient had no associated systemic risk factors. As the thinning increased, cyanoacrylate glue with bandage contact lens was applied, but the dellen reappeared seven days after glue removal. Dellen finally resolved with thermal punctal cautery applied to both puncta. Dellen most commonly respond to artificial tears, antibiotic ointment, and patch application. However, in refractory cases, punctal cauterization can be considered as a good option to increase tear pooling in the area of dellen, thereby promoting healing.

## Introduction

A pterygium is a fibrovascular tissue that often originates from the nasal bulbar conjunctiva and extends onto the cornea in a wing-shaped pattern [1]. Intraoperative antimetabolites, conjunctival limbal autograft (CLAG) transplantation, and amniotic membrane transplantation are the most used methods to decrease the rate of recurrence after surgery [2-6]. In CLAG transplantation, fibrin glue shortens the operative time and enhances postoperative comfort compared with the use of sutures [7].

Corneal dellen are small saucer-like excavations at the margin of the cornea that occur most often following the processes that produce a paralimbal elevation that can induce a localized break in the precorneal oily layer of the tears. This, in turn, causes localized dehydration and thinning of the cornea [8]. Overall, corneal dellen usually heal within 10-15 days [8-9]. Treatment consists of artificial tears, eye ointment, and patching. Bandage contact lens application has also been used to support the repair process [10]. If left untreated, this condition may lead to corneal perforation.

We report a case of corneal dellen that occurred 15 days after uneventful pterygium excision with CLAG with fibrin glue without antimetabolites and was treated successfully with punctal cautery.

## Case presentation

A 45-year-old man presented to us with growth and foreign body sensation in his right eye. On examination, his best-corrected visual acuity was 20/20 in both eyes. Slit-lamp examination of the right eye showed nasal pterygium on the cornea approximately 3 mm from the limbus, with scarring at its margins. In both eyes, tear film height was adequate, and the ocular surface was moist. The rest of the examination was unremarkable. His ocular and medical history were unremarkable.

**Figure 1:**
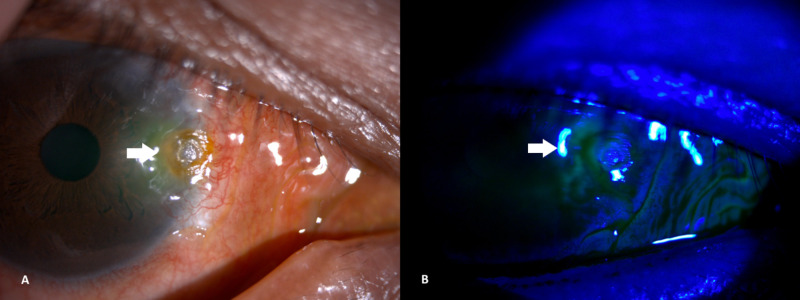
(A) Corneal dellen at the onset. (B) Corneal dellen - no fluorescein staining seen under cobalt blue light of slit lamp.

The patient underwent right eye pterygium excision with CLAG with fibrin glue (TISSEEL Lyo, Two-Component Fibrin Sealant Vapor heated, Baxter AG, Austria) under peribulbar anesthesia. Postoperatively, he was started on prednisolone acetate eye drops 1% four times daily, carboxymethyl cellulose 0.5% eye drops four times daily, and ciprofloxacin 0.3% eye drops four times daily, and was advised to apply a patch in between topical treatments. There were no concerns or complications noted for the first 15 postoperative days. On postoperative day 16, the patient presented with nasal corneal dellen (Figure 1).

**Figure 2:**
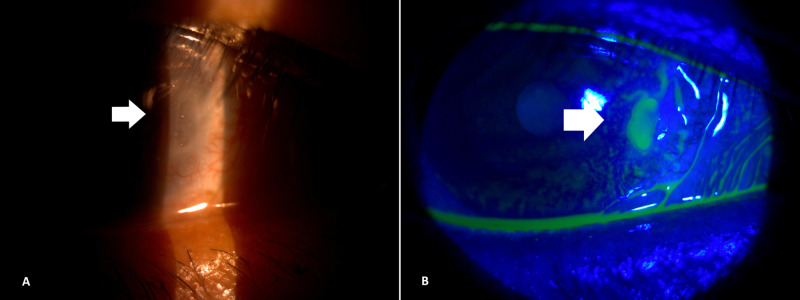
(A) Reappearance of corneal dellen after removal of cyanoacrylate glue and Bandage contact lens. (B) Pooling of fluorescein dye seen under cobalt blue light of slit lamp.

The steroid drops were stopped, lubricants were increased to an hourly rate, and he was advised to apply a patch in between topicals. Two days later, corneal thinning increased, and he underwent cyanoacrylate glue with bandage contact lens under topical anesthesia. Four weeks after the cyanoacrylate glue procedure, the glue and bandage contact lens were removed, and the dellen was found to have healed. Topical steroids were restarted four times a day, along with antibiotic eye drops four times a day, and lubricants two hourly. One week later, however, the dellen reappeared (Figure 2).

**Figure 3:**
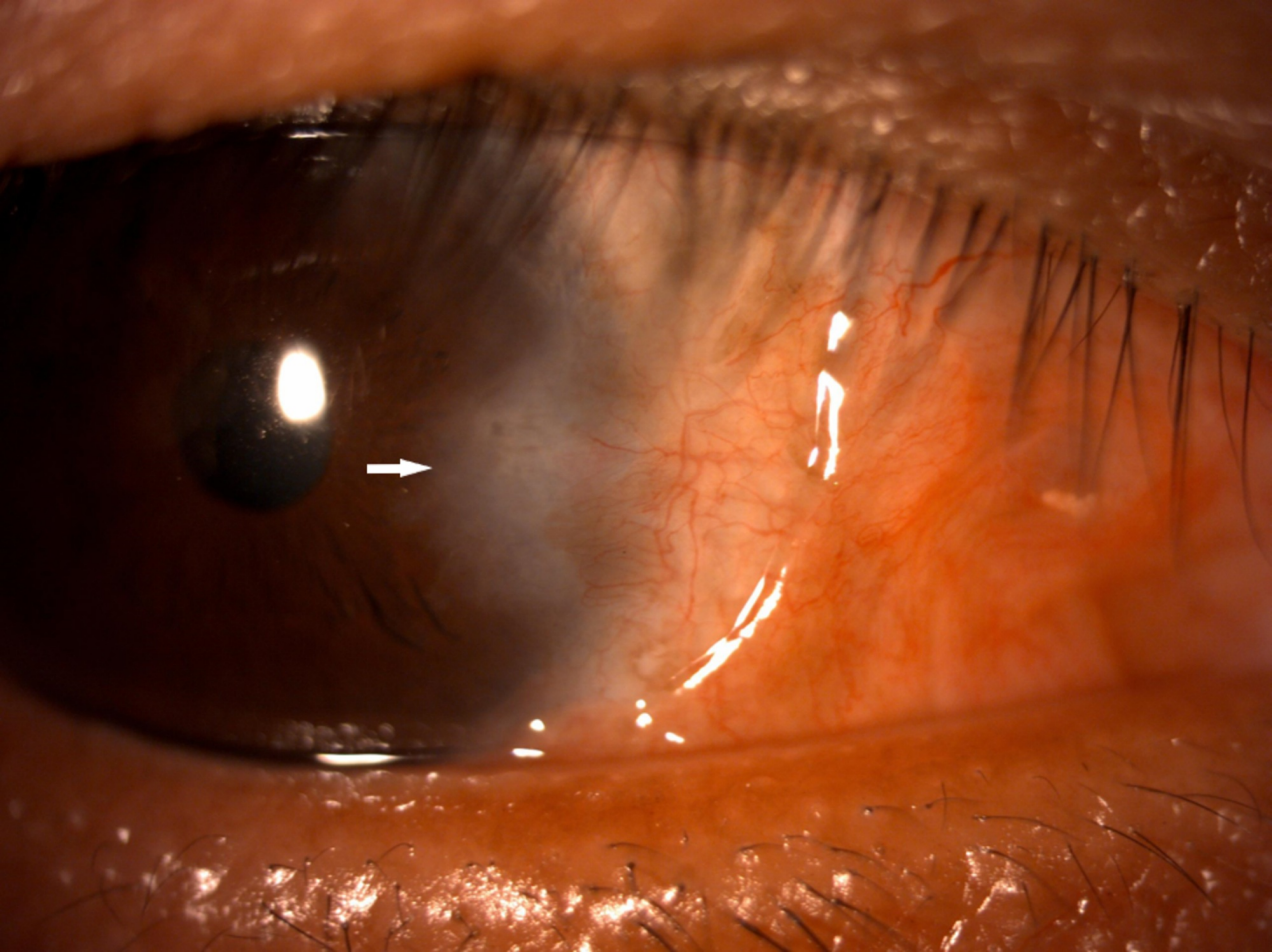
Corneal scarring nasally after two years of surgery.

Topical steroids were stopped; lubricants and antibiotics were continued. A systemic workup showed no underlying connective tissue disorder. Slit-lamp examination revealed poor tear flow to the area of dellen. This was attributed to mild graft edema nasally, which prevents tears from reaching the nasal cornea over the dellen. Option of temporary punctal plugs was discussed with the patient, but the patient refused the same for monetary reasons. Thermal punctal cautery was then applied under topical anesthesia to both the upper and lower puncta to achieve punctal occlusion. After five days, the dellen resolved with complete healing. Two years after the surgery, eye examination showed nasal corneal scarring with no recurrence without much epiphora (Figure 3).

## Discussion

Dellen are caused by interruptions of the tear film and local dehydration of the cornea. They were first described in 1911 as forming a saucer-shaped excavation of the cornea that typically occurs adjacent to a limbal elevation [11]. In the ophthalmic literature, dellen have been reported after pterygium surgery, large filtration blebs, dermoids, scleritis, episcleritis, scarring after extraocular muscle surgery, or severe conjunctival chemosis [9, 12].

Corneal dellen after pterygium surgery is a rarely reported complication regardless of the use of adjunctive therapy [8, 12-13]. Accorinti et al., described a patient with corneal and scleral dellen after a bare scleral technique without the use of antimetabolites and after a febrile episode, treated with topical lubricants and antibiotic eye ointment [8]. Tsai et al. [12] reported a case of scleral dellen as a complication of uneventful pterygium surgery performed with mitomycin C and then treated with topical lubricant therapy.

Gokhale reported a case of peripheral ulcerative keratitis after pterygium surgery without the use of mitomycin C, treated with cyanoacrylate glue [14]. Chen and Noonan reported a case of scleral dellen treated with a conjunctival flap and regular antibiotic ointment as a complication of a bare sclera excision without adjunctive therapy [15].

In the case described here, no sutures and no adjunctive substance like mitomycin C were used; there was also no bare sclera left during the surgery that might have predisposed to dellen formation. Although cyanoacrylate glue helped in healing the dellen, it failed to prevent its recurrence. Our patient exhibited no other ocular or systemic predisposing risk factors to ulceration, as his immunological workup was normal and remained so after two years of follow-up.

In our patient, persistent graft edema nasally may have caused paralimbal elevation, inhibiting normal tear distribution over the cornea. Graft edema is common after the use of fibrin glue during pterygium surgery due to its antigenic components. This was most likely the promoting factor favoring the onset of the corneal dellen. The dellen rapidly improved after thermal punctal cautery was applied to both the upper and lower puncta, supplemented with tear-film substitutes, antibiotic eye drops, and patching.

To the best of our knowledge, this represents the first reported case of dellen after pterygium excision with CLAG with fibrin glue, without any adjuvant treatment that healed with thermal punctal cauterization.

## Conclusions

Corneal dellen might occur after uneventful pterygium surgery with CLAG with fibrin glue, performed without adjunctive therapy. Postoperative persistent graft edema might inhibit proper tear distribution over the cornea and hence predispose to corneal dellen formation. Punctal cauterization may be used effectively to increase tear pooling over the affected area along with copious lubricant application for early resolution of dellen, thereby preventing corneal perforation.
